# Radiation exposure facilities for radiobiology in Europe: availability, capabilities, and limitations

**DOI:** 10.1007/s00411-025-01173-9

**Published:** 2025-11-22

**Authors:** Tom Unterleiter, Maya Shariff, Michael Rückert, Lena Winterling, Laura Ruspeckhofer, Thomas Weissmann, Florian Putz, Rainer Fietkau, Christoph Bert, Udo S. Gaipl, Lisa Deloch

**Affiliations:** 1 Department of Radiation Oncology, Universitätsklinikum Erlangen, Friedrich-Alexander-Universität Erlangen-Nürnberg, Erlangen, Germany; 2 Translational Radiobiology, Department of Radiation Oncology, Universitätsklinikum Erlangen, Friedrich- Alexander-Universität Erlangen-Nürnberg, Erlangen, Germany

**Keywords:** Radiation protection, Experimental setups, Mixed-Beam, Alpha-Radiation, Radon, Radiation research

## Abstract

Experimental radiobiology studies rely on exposure platforms that replicate real-world scenarios, yet information on their availability and biological suitability is often fragmented. We thus aimed to map capabilities, access routes, and limitations of European irradiation facilities, with a focus on Germany and briefly contextualized it with selected platforms in the United States of America (U.S.). Single-source systems (X-ray, gamma, alpha/beta) are widely available for cell/animal work, but mixed-beam platforms with controlled conditions and traceable dosimetry are scarce and oversubscribed while alpha/radon analogue setups vary in geometry and atmosphere control, hindering comparability. Large user facilities (reactors, cyclotrons, space-simulation) offer powerful modalities but often lack clearly documented biological access procedures. Here, the selected U.S. facilities provide more explicit user pathways (proposal, fee-for-service, programmatic access). Priorities for Europe should thus include standardized, incubator-compatible mixed-beam systems; a more harmonized reporting of dosimetry/environmental parameters; and a better visibility of biological access in infrastructure catalogues. Ongoing coordination initiatives can underpin these improvements, strengthening reproducibility and access.

## Introduction

Radiation exposure is a constant and often individualized aspect of daily life, resulting from both natural and artificial radiation sources such as cosmic background radiation, radioactive materials in soil, and man-made radiation from medical procedures and industry (Shahbazi-Gahrouei et al. 2013). To ensure public safety, comprehensive radiation protection laws and guidelines have been established throughout the world. Current European regulations prescribe an average annual effective dose limit of 1 mSv for the general public, with an actual background population average of about 3–4 mSv/y, and an occupational exposure limit of 20 mSv per year (which in some circumstances can reach 50 mSv for a single year provided the average over five years does not exceed 20 mSv). For workers routinely exposed to ionizing radiation (IR), the effective dose must not exceed 100 mSv within five consecutive years (Strengthening Radiation Protection in EU; Directive 2013/59/Euratom). Maintaining expertise and infrastructure for radiation research remains crucial despite reductions in specialized facilities and challenges in funding (Rühm et al. 2023). Controlled studies of biological effects of IR, particularly with varying radiation qualities, are essential for optimizing risk assessment, protection, and prevention. Differences in the impact of alpha particles, beta particles, or photons on biomolecules and repair processes highlight the need for access to dedicated experimental platforms capable of delivering multiple radiation qualities (Barendsen 1979; Cary et al. 2012). Such platforms are vital for modeling exposure scenarios and understanding both immediate and delayed cellular responses, which may differ based on age, sex, and pre-existing medical conditions (Deloch et al. 2022; Völlings et al. 2022). Notably, recent European initiatives—such as MELODI, CONCERT, PIANOFORTE, and others—have worked to consolidate, document, and promote research infrastructure for radiation research, including exposure platforms. These coordinated efforts respond to the need for improved accessibility and cooperation across Europe. Despite these advances, significant challenges remain in transparent documentation and broad availability of irradiation platforms, especially for radiobiology studies and even the most recent catalogue SNETP does not consistently list radiobiology-dedicated assets (Air2D2, 2025; Kreuzer et al. 2018a; PIANOFORTE 2024; SNETP 2025).

This review incorporates up-to-date information on radiation exposure facilities for radiobiology studies. The ultimate goal is to support continued excellence in research and radiation protection policy by critically evaluating the current landscape and persistent challenges, with focus on Europe. This review intentionally limits general information to retain a focused scope and the central aim is to provide an overview and critical assessment of experimental irradiation platforms available for radiobiology and radiation protection research, with a particular emphasis on facilities in Europe and especially Germany. The review does not attempt a comprehensive analysis of molecular radiation effects; rather, it identifies the advantages and disadvantages of exposure facilities relevant for conducting biological research and highlights persistent infrastructure and expertise gaps. Although the primary focus of this review is on European facilities, radiation research is inherently international. Exposure scenarios, standards, and research infrastructures are shaped not only by regional regulations but also by global challenges such as aviation, space travel, nuclear safety, and medical applications, which often transcend national borders. Given the high degree of global scientific collaboration, and the fact that the United States of America (U.S.) hosts several well-documented and accessible irradiation platforms, introducing a broader perspective helps to identify best practices, highlight structural differences in accessibility, and reveal opportunities for international alignment and cooperation.

## Methods

A comprehensive literature and database search was conducted to identify and summarize irradiation platforms relevant for radiobiology and radiation protection research in Europe, with additional comparison to facilities in the U.S. Peer-reviewed articles, technical reports, and institutional sources were identified through searches on major databases including PubMed, Web of Science, Scopus, and Google Scholar. Specific attention was given to papers published in the last two decades describing technical characteristics, accessibility, and biological suitability of exposure facilities to increase chances of viability of included facilities. Additional facility and infrastructure data were compiled from European research networks and initiatives such as MELODI (https://melodi-online.eu), CONCERT (https://cordis.europa.eu/project/id/662287), PIANOFORTE (https://pianoforte-partnership.eu) and AIR2D2 (https://www.concert-infrastructures.eu). Institutional websites and official registries such as the IAEA Research Reactor Database (https://nucleus.iaea.org/rrdb/home) and SNETP (https://snetp.eu) were used to confirm operational status and capabilities. All literature was screened for relevance to biological and radiation protection research; only publications providing technical, dose-rate or institutional availability information were included for detailed summary. Limitations and access notes were documented where information was available.

### Alpha particles

Alpha particles are doubly charged helium nuclei (He²⁺) which deposit energy along extremely short trajectories (approximately 4 cm in air), resulting in a localised high linear energy transfer (LET). For example, a 2.5 MeV alpha particle has an LET of around 166 keV/µm in the Bragg peak, which far exceeds the value for X-rays (250 kVp: ~2.0 keV/µm) (Hall and Giaccia 2011). This high LET poses a significant challenge for in vitro irradiation and spatial mapping in vivo is generally infeasible. Radon (and its decay products) is the main natural source of alpha particles that is relevant in the context of radiation protection and radiobiological research (Deloch et al. 2022; Paz et al. 2021; Rühle et al. 2017). According to the World Health Organization (WHO), radon is the second leading cause of lung cancer (3–14% of cases) and can account for up to one third of the annual radiation dose received by some populations (*World Health Organization: WHO Handbook on Indoor Radon: A Public Health Perspective. * 2009). Importantly, the dose to lung tissue is what matters for risk analysis, not the whole-body effective dose which, due to local energy deposition is very low (United Nations Scientific Committee on the Effects of Atomic Radiation, 2020). Typical residential radon concentrations range from 20 to 300 Bq/m³, but occupational environments (e.g. mines) can reach millions of Bq/m³ (ICRP 2017; Kreuzer et al. 2018b; Madas et al. 2022). Estimated annual alpha particle doses to lung tissue are in the region of several mGy in residential settings and in the region of tens to hundreds of mGy in workers exposed to radon in the course of their employment (Beck 2024). Monte Carlo simulations have provided detailed estimates of lung tissue doses, bridging the gap between radon concentrations and biological damage. Abu Shqair and Kim (2021) calculated tissue doses ranging from 0.85 Gy for secretory cells to several Gy for other cell types during occupational exposure. Tissue dose increases by 7.17 Gy for each unit of alpha-emission density. During 8-hour shifts in mining conditions, the bronchial epithelium receives an approximate total lung dose of 5.29 Gy, with ⁹²⁸Po contributing 92.8% and ²¹⁰Po 7.2% (Abu Shqair and Kim 2021). These computational estimates provide crucial validation data for experimental radon exposure facilities.

The available experimental platforms in Europe vary substantially in their ability to simulate real exposure scenarios. For instance, the Federal Office for Radiation Protection in Germany (BfS) runs a radon chamber research facility with adjustable radon concentrations ranging from 50 to 50,000 Bq/m³ and controllable environmental parameters (Hamel and Schmidt 2001). The GSI Helmholtz Centre in Darmstadt has a 50 L chamber with a 226Ra source (up to ~ 640 kBq/m³) surrounded by a climate-controlled water bath (Maier et al. 2015). Other facilities in Paris (ASNR) and Berlin (BfS) are used to calibrate measurement devices and can be adapted for biological research.

In general, radon exposure of cells can be carried out via a radon-saturated medium or direct atmospheric exposure. ²²RnCl₂ is commonly used for therapeutic exposures, but has also been utilised for in vitro radiobiological experiments (Schumann et al. 2018). In radiobiology studies, alpha particle exposure is typically performed using specialised setups designed to ensure the controlled irradiation of in vitro samples. The main approaches are summarised in the table below and include radon chambers, planar alpha sources and the use of alpha-emitting isotopes in solution. Each approach presents unique technical challenges and offers potential for precise dosimetry, sample placement and reproducibility. In vitro alpha particle experiments typically employ planar sources (e.g. ^241^Am, ^238^Pu and ^239^Pu) to mimic exposure, often achieving dose rates ranging from a few mGy/min to several Gy/min depending on the geometry and strength of the source (Maier et al. 2020; Roos and Kellerer 1989). An overview of selected facilities using alpha particles is given in Table 1.

**Table 1:** – Selection of facilities using alpha particles with their location, dose rate and technical details

Authors	Setup/Source	Institution (Type)	Dose Rate (Gy/min)	Technical Notes/Activity and Country
(Petitot et al. 2002)	Radon Chamber, ^226^Ra (30 kBq/m³)	IRSN, Paris (Res. Inst.)	n/a	Climate control, France
(Maier et al. 2015)	Radon Chamber, ^226^Ra (up to 640 kBq/m³)	GSI, Darmstadt (Res. Inst.)	n/a	Water bath, CO₂/humidity control, Germany
(Hamel and Schmidt 2001)	Radon Chamber, ^226^Ra (50–50,000 Bq/m³)	BfS, Berlin (Res. Inst.)	n/a	Accredited, aerosol/climate control, Germany
(Purrott et al. 1980)	Alpha particles, ^239^Pu	Univ. Oxford (Univ.)	~ 1	Custom chamber, England
(Nikitaki et al. 2021)	Alpha particles, ^234^U	Univ. Athens (Univ.)	16–40 min for 1 Gy, depending on setup	Custom device, Greece 4.9 MeV
(Goodhead et al. 1991)	Alpha particles, ^238^Pu	NRPB, Chilton (Res. Inst.) England	2 Gy/min – 10^− 4^ Gy/min or 24 Gy/min	Versatile LETs (0.8 to 4.2 MeV and 266 to 102 keV microns^− 1^), Helium chamber
(Simmons et al. 1996)	Alpha particles, ^238^Pu	St Thomas’/Guy’s Hospital, London (Hosp.)	n/a	Lung/hamster cell studies, England
(Hakanen et al. 2006)	Alpha particles, ^238^Pu	STUK, Helsinki (Res. Inst.)	n/a	Dosimetry/spectrum measurements, Finland
(Tisnek et al. 2009)	Alpha particles, ^238^Pu	Univ. Oslo (Univ.)	1.2 Gy/s	Irradiator for cultured cells, Norway, Helium tube, 5.5 MeV, 1.3GBq
(Roos and Kellerer 1989)	Alpha particles, ^241^Am 370MBq	Univ. Würzburg (Univ.)	0.2 Gy/min	Custom geometry, Germany
(Maier et al. 2020)	Alpha particles, ^241^Am 40MBq	GSI, Darmstadt (Res. Inst.)	8.2 ± 2.4	2.7 mm cell-source gap, Germany
(Moreira et al. 2021)	Alpha particles, ^241^Am 7.4MBq	Queen’s Univ. Belfast (Univ.)	1.57 Gy/min	Custom design/dosimetry, Northern Ireland, UK
(Staaf et al. 2012)	Mixed X-ray/^241^Am, ~ 50 MBq, 190 kVp X-ray)	Univ. Stockholm (Univ.)	Alpha 0.229 Gy/min 190 kVp X-ray)	Mixed beam, Sweden
(Phoenix et al. 2009)	Mixed gamma ^60^Co/^238^Pu (alpha), helium chamber	Univ. Oxford (Univ.)	Variable 25 Gy/min – 0.15 Gy/min High LET 50 Gy/min – 0.001 Gy/min Low LET	Mixed beam, England

### Beta particles

Although less common than alpha or photon radiation in environmental and occupational exposure, beta radiation is still relevant, especially in nuclear power operations and medical use. Notable beta emitters include iodine-131, yttrium-90 and rhenium-188, which are found in fission products and are frequently used in therapy and diagnostics. Beta particles (electrons or positrons) are emitted from nuclei with excess neutrons. They are much less ionising than alpha particles and can travel tens of centimetres in air and up to several millimetres in tissue, reaching greater penetration depths (Muller 1957). The Beta Secondary Standard BSS 2 (Eckert & Ziegler, Germany) is a widely used beta research and calibration device, primarily used for dosimeter calibration, but it also offers precise control over activity and environmental parameters and is potentially suitable for biological exposure studies (Behrens and Buchholz 2011). In research, radioimmunoconjugates (e.g. ⁹⁰Y-labelled compounds) have been used for in vitro cell irradiation in cancer studies (Friesen et al. 2003). The radioactive isotope tritium (³H) is sometimes used for cellular studies, but it poses significant dosimetry challenges and can lead to inhomogeneous exposure (Le et al. 2015).

### Photons

In contrast to the aforementioned radioactive particles, radiation in the form of photons is best described as an electromagnetic wave, and the degree of ionisation is determined by the energy of the photons. The spectrum ranges from non-ionising ultraviolet (UV) radiation, which has significant biological effects, to ionising X-rays and gamma rays. X-rays are produced in an atom’s electron shell, while gamma rays originate from the nucleus (Serway and Jewett 2004). Due to their commercial availability and capacity for rapid, relatively homogeneous exposures of biological samples, photon emission devices, especially X-ray generators, are widely used in biological radiation research (Samarth et al. 2023). Such devices are also predominant in medical radiotherapy treatments; (Healy et al. 2017).

However, recent literature highlights that achieving true homogeneity and precise dosimetry in photon irradiation is technically challenging. Many recent studies report that numerous setup components, such as beam collimation, sample geometry, dose measurement position, filtration and environmental conditions, must be carefully controlled to ensure reproducible and accurate irradiation, particularly in cell culture experiments. Incomplete reporting and insufficiently characterised setups have been shown to hinder replication and comparison across laboratories and can introduce systematic errors in dosimetry and estimation of the biological effect (Wojcik et al. 2024). These challenges have been particularly noted in multicentre or non-standard exposure studies, as emphasised in several recent methodological reviews and meta-analyses (Bucher et al. 2021, 2022; Claridge Mackonis et al. 2018; Desrosiers et al. 2013; Dos Santos et al. 2018, 2021; Dos Santos and Trompier 2025; Draeger et al. 2020; Kuess et al. 2014; Noblet et al. 2014; Trompier et al. 2024).

Thus, when summarising and comparing different photon irradiation platforms, it is crucial to consider the detailed reporting of all physical and dosimetric parameters, as recommended in recent consensus articles. The complexity and variability of laboratory setups mean that careful documentation is necessary to ensure that experiments can be reliably replicated and outcomes from different studies can be meaningfully compared.

## Mixed-field irradiation and special facilities

Mixed-field irradiation platforms, which enable exposure to various types of radiation such as alpha particles, X-rays, gamma rays, protons and neutrons, are becoming increasingly important in modern radiobiology. They allow researchers to recreate the complex mixtures of high- and low-linear energy transfer (LET) radiation encountered in occupational, environmental and clinical contexts. Depending on facility design, sample handling, and the nature of the biological question under study, both simultaneous and sequential irradiation approaches are employed. Careful consideration of dosimetry, environmental control, and the temporal sequence of exposures is required when designing assays, as biological effects frequently depend on these parameters and can yield additive, synergistic, or antagonistic outcomes (Guerra Liberal et al. 2023; Phoenix et al. 2009; Staaf et al. 2012). State-of-the-art laboratory setups demonstrate the technological diversity and experimental flexibility that is now possible. The MAX facility at Stockholm University, for example, can deliver simultaneous or sequential alpha and X-ray exposures to cell cultures under tightly regulated temperature and CO₂ conditions. Both track-etch and ionisation chamber dosimetry was used to to accurately characterise the LET and dose rates (Staaf et al. 2012). The Gray Institute in Oxford has a helium-filled irradiation chamber that combines a ^238^Pu alpha source with a ^60^Co gamma source. This allows for the careful staging of mixed-field experiments on biological monolayers (Phoenix et al. 2009). Experiments at Queen’s University Belfast alternate alpha, X-ray and proton irradiation among standard culture systems using rapid switching protocols that are well suited to mechanistic studies of DNA damage response and repair (Guerra Liberal et al. 2023). Access to mixed-beam possibilities is further broadened by larger European and national user facilities such as CERN, IRRAD, JSI, TRIGA Reactor, IFJ PAN Cyclotron, UCLouvain CRC and Birmingham MC40 Cyclotron. These facilities encompass heavy ions, high-energy neutrons and complex programmable exposures for advanced detector and radiobiology projects. They have been mentioned in the SNETP catalogue, but the availability of radiobiological facilities must be confirmed on site (SNETP 2025).

These research landscapes consist of specialised, multimodal research infrastructures spread across Europe. Originally established to support space research, materials testing or nuclear science, they now also enable nuanced biological and radiobiological experimentation. A prime example is the Complex Irradiation Facility (CIF) at DLR Bremen, where biological and material samples can be exposed simultaneously in an ultra-high vacuum to low-energy protons (1–100 keV), electrons and UV or X-ray electromagnetic sources across an 80 mm sample zone. The chamber provides precise thermal control (80 K to 450 °C) and programmable gas environments, enabling the simulation of extraterrestrial conditions or atmosphere-modified irradiation of living and non-living materials. While the CIF’s original focus was on space hardware, the system is open to biological and astrobiological studies when collaboration is arranged (Renger et al. 2014).

For astrobiology and planetary protection experiments, the *Deutsches Zentrum für Luft- und Raumfahrt* (German Aerospace Center, DLR) ABYSS and AstroBiologY space simulation facilities at the Institute of Aerospace Medicine offer full-spectrum irradiation, from vacuum and low pressures to high-temperature regimes, with modular UV, X-ray and particle sources. The facility is regularly used for eukaryotic and microbial radiobiology under atmospheric and space-relevant conditions (Jordan and Hemmersbach 2021), and can accommodate biological studies of bacteria, spores, single cells, and higher organisms. The ELBE electron accelerator at the Helmholtz-Zentrum Dresden-Rossendorf delivers pulsed or continuous electron beams (6–32 MeV, up to 1 mA), with the ability to switch between direct electron and Bremsstrahlung photon exposures. An on-site cell laboratory enables the irradiation of cell lines, tissues and 3D cultures, facilitating the development of FLASH and ultra-high dose rate assays as well as conventional low-dose radiobiology. The facility can also deliver neutrons and positrons for specialised projects (Karsch et al. 2022). Large-scale sources, such as the ISIS Neutron and Muon Source (UK) and the GELINA/JRC EUFRAT facility in Belgium, offer pulsed neutron, muon or gamma irradiation to biological or material targets. Their open, proposal-driven research programmes allow biosciences researchers to apply for beamtime and accommodate assay types ranging from single-cell genomics to ecosystem-level dosimetry. These facilities further strengthen Europe’s infrastructure for interdisciplinary radiobiology, serving as key links between health, nuclear, and fundamental physics research (Commission 2024).

Overall, the combination of specialised mixed-beam irradiation platforms and large-scale facilities enables radiobiological researchers in Europe to address complex real-world exposure scenarios that cannot be replicated using single radiation sources. However, although there is an extensive network of irradiation facilities across Europe, the most recent infrastructure registries, such as the SNETP catalogue, focus primarily on technical and physical aspects and rarely address suitability or established procedures for biological experiments. This makes it difficult for radiobiologists to ascertain whether and how biological assays can be accommodated or if proposals involving living samples are accepted. Consequently, information about access for biological research is often difficult to find before contacting the facility. Improving the visibility and accessibility of biological capabilities at these facilities is essential for supporting innovative radiobiological research in Europe. An overview of selected mixed-beam as well as modular facilities is shown in Table 2.

**Table 2:** Overview of a selection of Mixed-Beam and modular facilities with their location and technical details

Facility/Publication & Location	Radiation Types/Source	Dosimetric Characteristics	Cell/Sample Holder & Conditions	Exposure Mode	Technical Notes/Access	Reference
Stockholm Univ. MAX	^241^Am alpha (planar, ~ 50 MBq); X-ray tube (190 kVp)	Alpha: ~0.2–0.24 Gy/min, LET 100–172 keV/µm; X-ray: 0.05–0.07 Gy/min	Polyamide disk + Mylar foil in incubator, 37 °C, 5% CO₂	Simultaneous/ Sequential	Simultaneous delivery, PADC track-etch, ion chamber dosimetry; angular alpha spread, open to research	(Staaf et al. 2012)
Gray Inst. Oxford	^238^Pu alpha (planar); ^60^Co gamma	Alpha LET ≈ 100 keV/µm; Gamma 1.25 MeV, std rates	Thin Mylar in helium atmosphere	Sequential/Simultaneous	Helium to reduce energy loss; separate sources; thorough dosimetric calibration; user facility	(Phoenix et al. 2009)
Queen’s Univ. Belfast	^241^Am alpha/proton cyclotron/X-RAD 225 kVp X-ray	Alpha: 1.57 Gy/min, LET 129 keV/µm; X-ray: 0.59 Gy/min, 2 mm Cu; Proton: 1, 12 keV/µm, ~ 5 Gy/min	Standard cell culture plates	Sequential	X-RAD225, proton via cyclotron, rapid switching; in vitro; uses modern radiobiology dosimetry	(Guerra Liberal et al. 2023)
(USA, MCW)	^60^Co gamma (1.25 MeV); 15 MeV neutrons (D-T)	LET neutrons: 30–60 keV/µm; dose rates clinical range	Standard cell culture	Simultaneous/Sequential	D-T neutron generator and ^60^Co; switched by interleaving or overlap; full dosimetric mapping	(Higgins et al. 1983)
CERN IRRAD/GIF++ (Switzerland)	Proton, gamma	Proton: up to 24 GeV/c; gamma: ^137^Cs, 662 keV	Irradiation stations	Simultaneous/Sequential	Detector and cell irradiation; extensive dosimetry; access via proposal, Europe-wide	(SNETP 2025)
JSI TRIGA Reactor (Slovenia)	Gamma, neutron	Variable, up to research reactor class	In-reactor or sample station	Simultaneous	Reactor, user access for biology/detector studies; moderated beams	(SNETP 2025)
IFJ PAN Cyclotron (Poland)	Proton/alpha	10–60 MeV; flexible beam parameters	Sample holders for cells/tissue	Sequential/Simultaneous	Proton/alpha custom irradiation, biology/detector projects, high-precision dosimetry	(SNETP 2025)
UCLouvain CRC (Belgium)	Heavy ions, protons, photons	LETs from low to high, multi-energy	Custom chambers for cell lines	Flexible	Facility specialized for heavy ion and particle radiobiology	(SNETP 2025)
Birmingham MC40 Cyclotron (UK)	Proton, neutron	Proton up to 40 MeV, neutron generator	Radiobiology and detector chambers	Simultaneous/Sequential	Proton, neutron and mixed beam experiments; user access	(SNETP 2025)

### Radiation research facilities in the U.S

In the U.S. there is a wide range of research facilities available. Similar to Europe, a large number of linear accelerators (LINACs) are located in therapy facilities or clinics that are sometimes also used for research. Ballas et al. (Ballas et al. 2006) compiled a comprehensive list of 2,246 radiation facilities. The United States Environmental Protection Agency (EPA) and the United States government also provide an overview of radiation research facilities on their website (*National Laboratories*; *Research Centers*,* Programs*,* and Science Advisory Organizations*) (Research Centers 2024). The EPA also has its own laboratory: the National Analytical Radiation Environmental Laboratory (NAREL) (*About EPA’s National Analytical Radiation Environmental Laboratory (NAREL)*) (About EPA’s 2024).

A wide range of radiation facilities suitable for low-dose radiation research have been collected (National Academies of Sciences 2022). Among these facilities is the Armed Forces Radiobiology Research Institute (AFRRI), which allows outside researchers to access its facilities after submitting a proposal. AFRRI offers a TRIGA (Training, Research, Isotope, General Atomics) Mark-F nuclear research reactor. This reactor can administer a mixed field of gamma and neutron radiation. The facility also contains high- and low-level cobalt-60 facilities, a LINAC and a small animal radiation research platform (SARRP) (National Academies of Sciences, 2022; *Radiation Facilities*). Colorado State University’s radiation facilities are open to external researchers on a fee-for-service or collaborative basis. They offer two low-dose-rate gamma-ray tissue culture facilities, one low-dose-rate neutron tissue culture facility and one low-dose-rate gamma-ray and neutron vivarium for small animal exposure (Borak et al. 2021; Kato et al. 2007; National Academies of Sciences, 2022; Shakhov et al. 2012; Wilson et al. 2008). The Loma Linda University James M. Slater, M.D. Proton Treatment and Research Center has a wide range of radiation facilities which are also open to external researchers free of charge. However, these facilities have limited capacity and require approval by a research committee. They offer both proton and photon (LINAC) radiation sources (National Academies of Sciences, 2022). The NASA Space Radiation Laboratory at Brookhaven National Laboratory is open to other researchers under a Strategic Partnership Project (*Research Partnerships and Technology Transfer*,* Brookhaven National Laboratory*). The facility has access to proton and electron beam ion sources, as well as a caesium-137 gamma-ray source (*Brookhaven National Laboratory*,* NASA Space Radiation Laboratory*; National Academies of Sciences, 2022; Tessa et al. 2016). It also enables galactic cosmic ray simulation using a spectrum of ion beams consisting of protons, helium ions and heavier ions (Norbury et al. 2016; Simonsen et al. 2020). The Columbia University Radiological Research Accelerator Facility (RARAF) uses charged particle and neutron beams. Some of these beams are specifically designed for low-dose radiation research, e.g. the microbeam beamline (singletron particle accelerator), which delivers one particle to targeted cells and is used to simulate the effects of background radon exposure. This accelerator generates ion beams, including protons, helium ions (alpha particles), lithium ions, boron ions, and carbon ions (Randers-Pehrson et al. 2009). The VAriable Dose-rate External 137Cs Irradiator (VADER) uses 137Cs brachytherapy seeds to expose animals and cell cultures (Garty et al. 2020; National Academies of Sciences, 2022). They also provide a clinical accelerator adapted to deliver ultra-high doses (FLASH) to mimic exposure to nuclear events similar to those in Hiroshima and Nagasaki (Grilj et al. 2020; National Academies of Sciences 2022), as well as an accelerator-based neutron source using the Hiroshima atomic bomb as an example of the spectrum output (mixed proton/deuteron beam on a beryllium target). This can be used to examine nuclear disasters and space radiation, such as lunar albedo neutrons (National Academies of Sciences, 2022; Xu et al. 2015). These facilities can be used by external researchers. For radon research, the Radon Outreach and Research (ROAR) Project at the University of North Dakota was one of the few research facilities planning to build a radon exposure chamber (*Radon Outreach And Research (ROAR)*,* University of North Dakota*).

## Discussion

Whether occupational, environmental or medical, radiation exposure scenarios, they all require experimental platforms that can accurately emulate real-world conditions in order to provide meaningful insights for radiation protection policy. Carrying out controlled studies of biological effects across different types of radiation remains essential for optimised risk assessment, protection and prevention strategies. This is particularly important given that exposure effects can vary significantly depending on individual factors such as age, sex, and pre-existing medical conditions (Deloch et al. 2022; Völlings et al. 2022).

Recent European initiatives have made significant progress in consolidating existing radiation research infrastructure: The MELODI platform, established in 2010, has developed strategic research agendas for low-dose radiation risk research across Europe. The CONCERT programme (2015–2020) successfully integrated European and national research programmes, and the current PIANOFORTE partnership (2022–2029), with 108 partners across 23 countries, continues this effort by focusing on radiation protection research coordination and infrastructure sharing. These collaborative efforts, along with the AIR2D2 database, represent substantial progress in documenting and promoting European radiation research facilities, thereby addressing previous gaps in infrastructure visibility and accessibility (Air2D2, 2025; Kreuzer et al. 2018a; PIANOFORTE 2024). Despite these advances, there are still significant challenges in terms of accessibility of facilities and documentation of biological suitability. While there are extensive networks of specialised irradiation facilities across Europe, many infrastructure registries focus primarily on technical and physical aspects, rarely addressing established procedures for biological experiments or accessibility criteria for radiobiology researchers. This is in stark contrast to the situation in the U.S., where facilities are generally well documented with clear biological research capabilities and access procedures (National Academies of Sciences, 2022).

Our analysis identified different categories of radiation exposure facility, each with distinct advantages and limitations for radiobiology research. Highly specialised multi-modal facilities, such as research reactors, accelerators and space simulation facilities, offer exceptional capability to emulate complex exposure conditions with high fidelity. Facilities such as the Complex Irradiation Facility (CIF) at DLR Bremen, the ELBE electron accelerator and various European cyclotrons can deliver sophisticated mixed-field exposures that closely mimic real-world scenarios, such as cosmic radiation during space travel or conditions in the event of a nuclear accident (Karsch et al. 2022; Pihet et al. 1994; Renger et al. 2014). However, these facilities face significant accessibility constraints, including limited beam times, high demand, competitive access procedures and often unclear policies for accommodating biological research.

In contrast, single-source radiation facilities, including commercial X-ray machines, linear accelerators used in clinical settings and dedicated isotope sources, offer excellent availability and accessibility. These systems enable a wide range of experiments, from basic research to specialised applications, and are relatively straightforward to operate and maintain. However, they are limited in their ability to emulate mixed-field exposure scenarios, which are common in occupational and environmental settings. Examples include exposure of aircraft personnel or complex environmental radiation conditions.

Mixed-field irradiation platforms represent a critical middle ground, but remain scarce across Europe. To our knowledge, based on published descriptions suitable for biological use, only two dedicated mixed-beam facilities were identified during our investigation. Stockholm University’s mixed-beam setup combines X-ray and alpha particle sources within an incubator environment, and the Gray Institute at Oxford operates a helium-filled chamber that combines alpha and gamma sources (Phoenix et al. 2009; Staaf et al. 2012). These platforms address the crucial need to study radiation quality interactions, as both additive and synergistic effects have been observed, depending on exposure conditions and sequence (Brooks et al. 1990; Furusawa et al. 2002; Higgins et al. 1983).

Alpha particle research faces particular challenges due to the unique properties; high LET and short penetration. Despite radon being identified as the second leading cause of lung cancer and a significant component of public radiation exposure, only two dedicated radon chambers for biological research were identified in Europe, supplemented by nine alpha particle analogue facilities using planar sources. The technical complexity of radon exposure systems, including the need for controlled atmospheres, precise dosimetry and sophisticated radiation protection measures, explains their limited availability (Maier et al. 2023). Each alpha exposure setup requires unique technical solutions and has its own limitations. While collimators improve the quality of the alpha particle beam, they reduce the particle flux and require careful positioning to prevent inhomogeneous exposure. Helium atmospheres reduce energy loss, but increase system complexity. Many existing setups, developed decades ago, require modernisation in the form of updated dosimetry systems and faster shutters to improve accuracy (Maier et al. 2020). These technical variations significantly complicate comparisons of results between laboratories and the reproducibility of studies.

The heterogeneity of experimental platforms and protocols identified in this review highlights the critical need for standardisation in radiobiology research infrastructure, particularly in terms of access and reproducibility. The lack of commercially available, standardised mixed-beam exposure systems particularly limits comparative studies and reproducibility of results. Furthermore, improved documentation of the biological research capabilities of existing facilities would greatly benefit the European radiobiology community. Recent publications have emphasised that achieving precise dosimetry and homogeneous irradiation, particularly with photon sources, requires careful control of numerous setup components, including beam collimation, sample geometry, dose measurement position, filtration and environmental conditions (Bucher et al. 2022; Dos Santos et al. 2018, 2021). Incomplete reporting and insufficiently characterised setups have been shown to hinder experimental replication and cross-laboratory comparisons, underscoring the importance of detailed technical documentation and standardised protocols.

The identified infrastructure gaps suggest several priority areas for the development of radiation research in Europe. Firstly, increasing the availability of mixed-beam exposure facilities would fulfil critical research requirements for occupational and environmental exposure scenarios. Secondly, the development of commercially available, standardised exposure systems would improve experimental reproducibility and facilitate cross-laboratory collaboration. Thirdly, improving the documentation of biological research capabilities within existing infrastructure registries would make facilities more accessible to radiobiology researchers. These infrastructures would support the quest for research needed to improve the system of radiological protection (Laurier et al. 2021).

The ongoing PIANOFORTE partnership provides an excellent framework for addressing these challenges through coordinated European efforts. By continuing to build on the foundations established by MELODI, CONCERT and related initiatives, European radiation protection research can maintain its scientific excellence while addressing persistent infrastructure and accessibility challenges (Air2D2, 2025; Kreuzer et al. 2018a; PIANOFORTE 2024).

## Conclusion

**Fig. 1:**
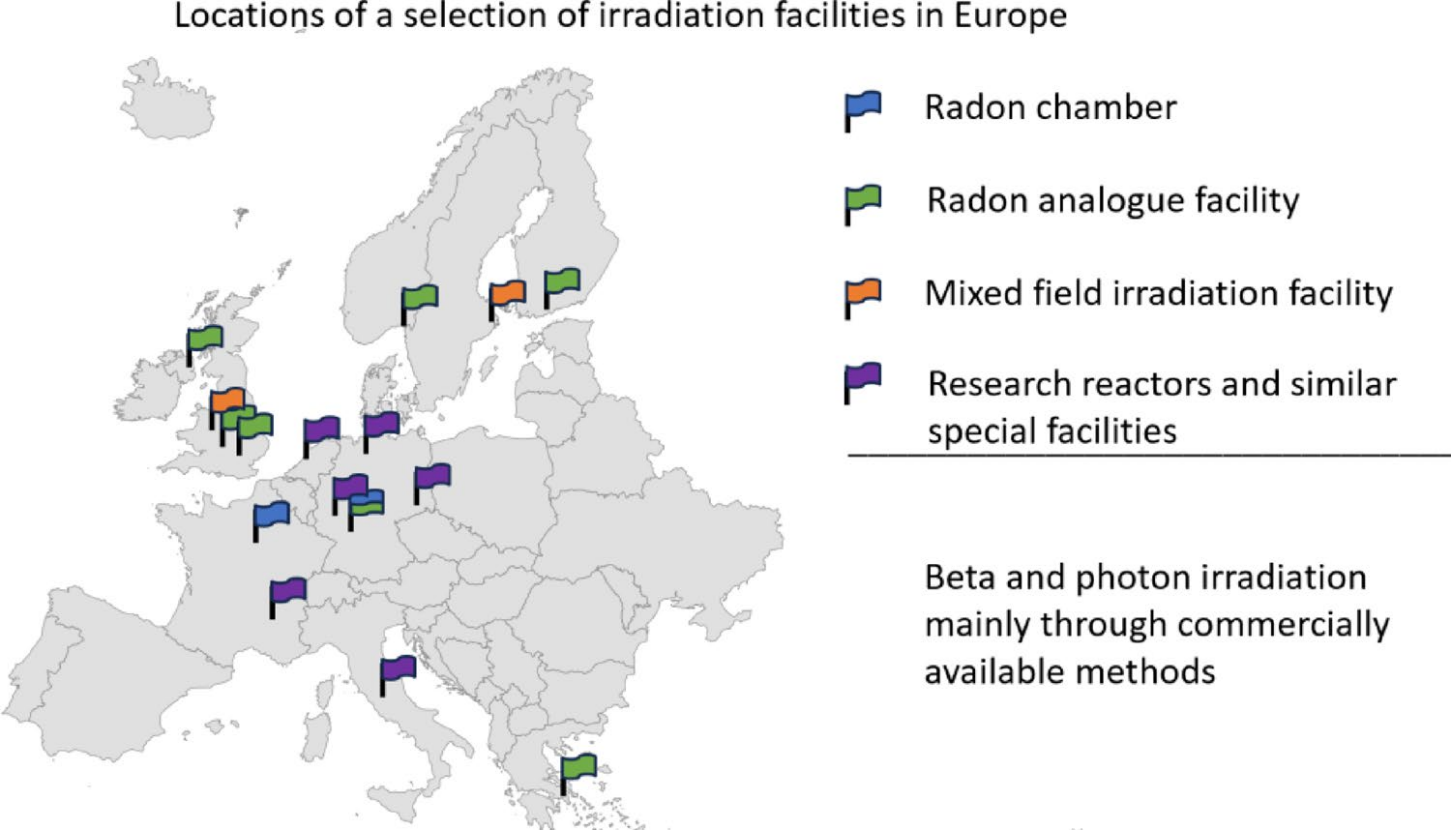
Overview of selected irradiation facilities in Germany and Europe: Radon exposure facilities are marked blue while radon analogue facilities are marked in green and reasearch reactors and specialized facilities are marked in purple. Beta- and photon-irradiation facilitues are not specified as they are usually available through commercial facilities or medical institutions.

Although Europe has a substantial radiation research infrastructure, improvements are needed in terms of access to facilities, standardisation and mixed-beam capabilities in order to fully support the complex research requirements of modern radiation protection and radiobiology studies. In order to gain better insights into how radiation affects health, the availability and viability of dedicated facilities for radiobiologic experiments is essential. This review provides an overview of existing exposure facilities to ensure that radiation protection-related knowledge remains up to date (Fig. 1).

## Data Availability

No datasets were generated or analysed during the current study.
